# Cold snaring for gastric sampling and for colonic adenoma resection: an ecological tip to use a single device for the whole endoscopy procedure

**DOI:** 10.1055/a-2067-4538

**Published:** 2023-04-26

**Authors:** Mathieu Pioche, Clara Yzet, Raphaelle Grau, Jean-Christophe Saurin, Mikael Mochet, Pierre Lafeuille, Jérôme Rivory

**Affiliations:** 1 Gastroenterology and Endoscopy Unit, Pavillon L, Edouard Herriot Hospital, Lyon, France; 2 Inserm U1032, Labtau, Lyon, France

The ecological impact of endoscopy is significant 1 and it is therefore crucial to seek strategies 2 3 for reducing the number of devices used and therefore the weight of waste generated 4 . Endoscopy procedures frequently involve both upper and lower gastrointestinal procedures, for example, gastric sampling for *Helicobacter pylori* and metaplasia detection on the one hand and colonoscopy with a high risk of detecting polyps on the other. Gastric sampling commonly involves the use of biopsy forceps. Nowadays, cold snaring is the reference method for removing small polyps (< 10 mm), which are very frequently detected during a screening colonoscopy. It means that two devices are usually used during a combined upper and lower gastrointestinal endoscopy procedure, even though cold snaring can perform gastric sampling adequately, and, in fact, provides larger samples with more tissue for pathologists to analyze.

We report on a patient with Biermer disease in whom gastric sampling in the fundus was required as well as colonoscopy follow-up of a previous adenoma resection. Gastric sampling was done with cold snaring (hybrid snare 15 mm; Olympus, Tokyo, Japan), as was resection of an adenoma detected in the sigmoid colon, following suction of the area to form a peduncle for snaring ( Fig. 1 , Video 1 ). By not using forceps, 72 g of forceps waste was avoided. Furthermore, biopsy forceps have a central needle, which carries a risk of needlestick injury for personnel; in France, this device is classified as waste from care activities with infectious risks and requires specific disposal management in a very high temperature oven, resulting in additional pollution.

**Fig. 1:**
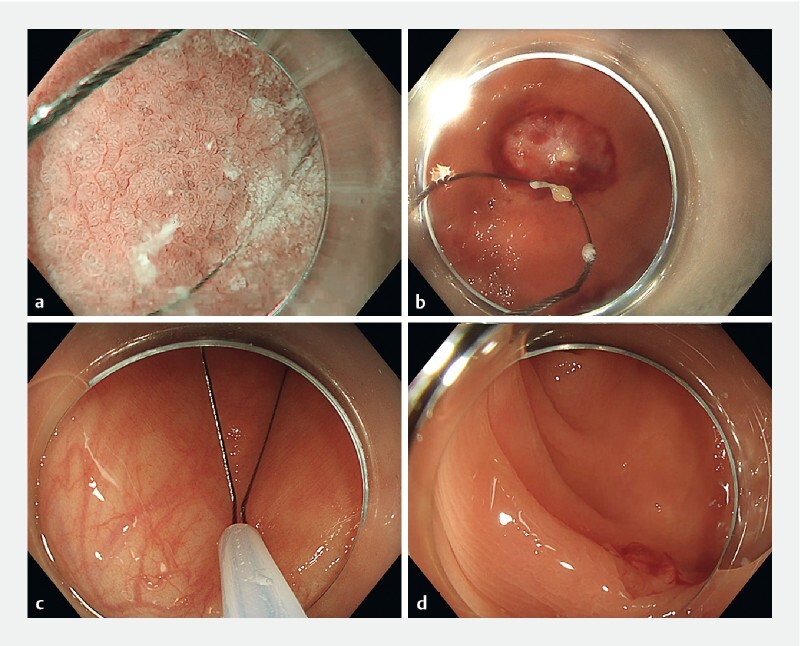
Double use of a single cold snare. **a** Gastric sampling with cold snare. **b** Area of resection in the stomach (large sample). **c** Colon diminutive polyp cold snaring. **d** Area of resection.

**Video 1** Cold snaring for gastric sampling and for colonic adenoma resection: use of a single device for the whole endoscopy procedure.


In conclusion, many devices can be used several times for different functions during an endoscopy procedure and therefore consideration of the whole procedure when planning resource use may reduce the need for some devices, and therefore reduce the cost and waste associated with our practices.

Endoscopy_UCTN_Code_TTT_1AO_2AC

## Notes

Endoscopy E-Videos

https://eref.thieme.de/e-videos
*E-Videos* is an open access online section of the journal *Endoscopy* , reporting on interesting cases and new techniques in gastroenterological endoscopy. All papers include a high-quality video and are published with a Creative Commons CC-BY license. Endoscopy E-Videos qualify for HINARI discounts and waivers and eligibility is automatically checked during the submission process. We grant 100% waivers to articles whose corresponding authors are based in Group A countries and 50% waivers to those who are based in Group B countries as classified by Research4Life (see: https://www.research4life.org/access/eligibility/ ). This section has its own submission website at https://mc.manuscriptcentral.com/e-videos
